# Return visit audits, quality improvement infrastructure, and a culture of safety: a theoretical model and practical assessment tool

**DOI:** 10.1007/s43678-023-00539-6

**Published:** 2023-06-15

**Authors:** Jesse T. T. McLaren, Tahara D. Bhate, Ahmed K. Taher, Lucas B. Chartier

**Affiliations:** 1 Emergency Department, University Health Network, Toronto, ON Canada; 2 Division of Emergency Medicine, Department of Family and Community Medicine, University of Toronto, Toronto, ON Canada; 3 Division of Emergency Medicine, Department of Medicine, University of Toronto, Toronto, ON Canada; 4 Toronto General Hospital, Toronto, ON Canada

**Keywords:** Return visit, Quality improvement

## Introduction

Return visit reviews have been a longstanding method of identifying emergency department (ED) adverse events and other quality issues, but the evolving culture of safety has shifted their focus: while the traditional view of “bouncebacks” reflected retrospective judgement on individual errors, “return visits” emphasize root-cause analysis and prospective change [1]. The field of quality improvement and patient safety (QIPS) within emergency medicine has developed significantly, but there is unevenness across national settings with gaps in infrastructure, training, and capacity [2].

Here, we describe our past 5 years of experience with QIPS from the local to the national level, and propose a theoretical model for understanding the interaction of safety culture, quality improvement (QI) infrastructure, and return visit audits. This forms the basis of an assessment tool, which should be helpful to EDs of any size in identifying next steps in their QIPS journey through the catalyst of return visit audits.

## A theoretical model for assessment and growth

**Fig. 1:**
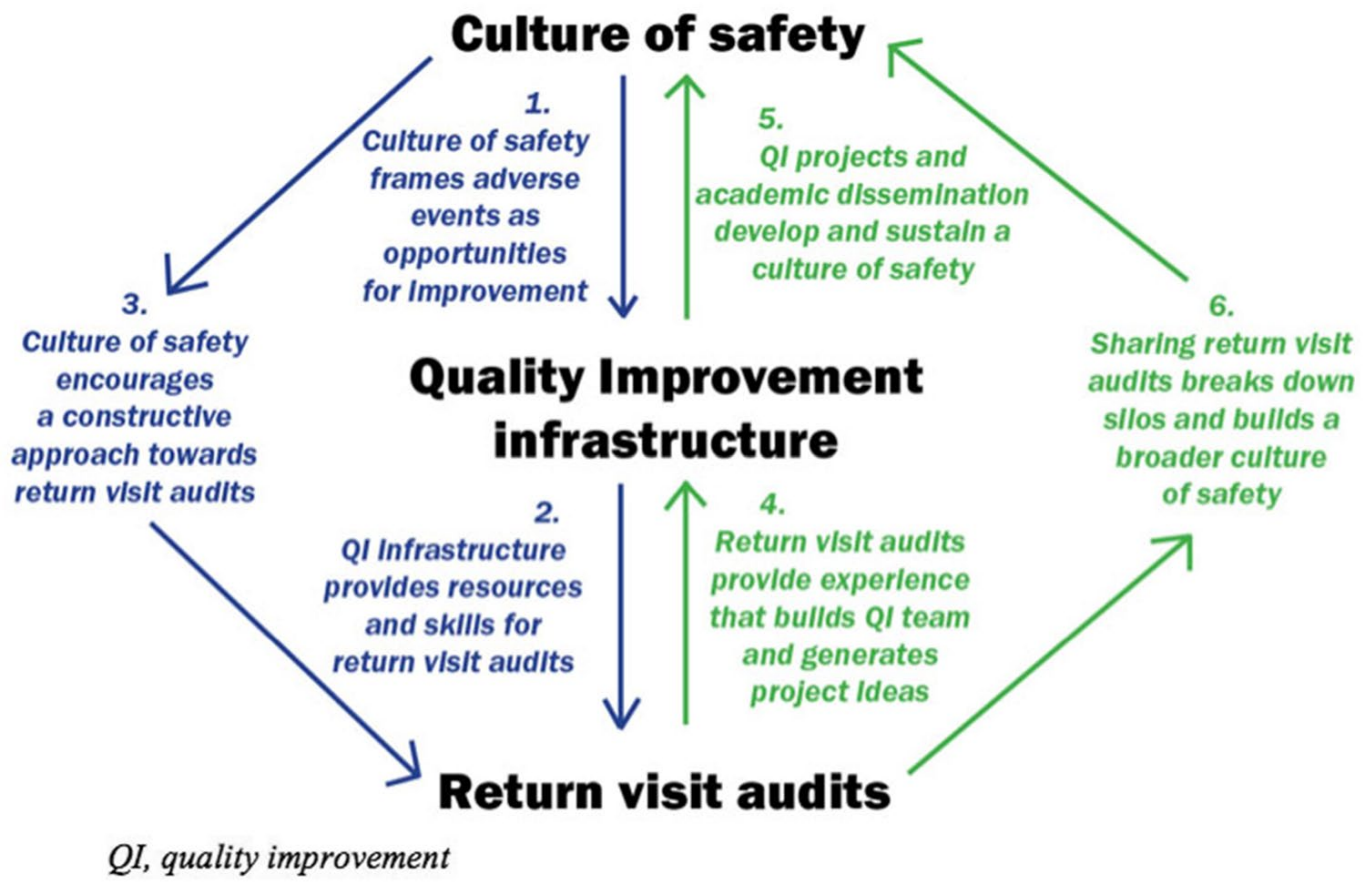
Theoretical model for mutual interactions between culture of safety, QI infrastructure and return visit audits

Healthcare QI strategies have often been described as “top-down” or “bottom-up”, with the former providing central coordination and resourcing, while the latter captures the ideas and commitment of frontline providers. However, this dichotomy fails to capture the complex relationships between safety culture, QI infrastructure, and return visit audits. Figure 1 provides a theoretical model for assessment and growth, including a combination of “top-down” interactions (the left half, in blue), and “bottom-up” interactions (the right-half, in green). These are not hierarchical or unidirectional, but cyclical and dynamic: each component is mutually dependent on the others, and each can be harnessed to bolster the others.

## Top-down

1. Culture of safety frames adverse events as opportunities for improvement.

A positive safety culture frames adverse events as opportunities for improvement, which encourages the growth of QIPS infrastructure. This varies widely: an environmental scan of emergency medicine academic centers across Canada found that 91% had developed QIPS committees but only 27.3% had administrative support and two-thirds had two or less physicians trained in QI [2]. Our center, the University Health Network (UHN), has a QIPS committee that includes more than 50 inter-professional team members—a dozen of whom are formally trained in QIPS. This partly reflects our resources as a tertiary academic center, but also reflects the deliberate application of safety culture: 7 years ago, we relaunched our QIPS committee, following a blueprint that can be replicated in other types of centers [3]. Analysis of return visits is one means by which a department can screen for and identify adverse events and other quality issues, in turn motivating QIPS infrastructure development.

2. Quality improvement infrastructure provides resources and skills for return visit audits.

QIPS infrastructure, both provincially and locally, influences the capacity for formal return visit audits. In 2016, the Ontario Ministry of Health and Long-Term care launched the ED Return Visit Quality Program (RVQP), mandating large hospitals (those 70 + hospitals with > 30,000 visits/year) to review ED return visits resulting in hospital admission. The RVQP is based on safety culture [4]: the goal is not to reduce return visits but to promote quality improvement. While there is no direct funding, the program provides return visit audit templates to identify root causes (patient factors, provider factors, and system factors). In its first 3 years, 86 hospitals conducted 12,852 return visit audits, uncovering 3,010 adverse events or quality issues, leading to hundreds of QIPS projects [5]. However, the quantity of chart audits ranged from a few to all return visits, and the quality ranged from a few sentences to full root-cause analyses. Further qualitative analysis found that this variation reflected pre-existing QIPS infrastructure, training, and support [6].

Leveraging our departmental QIPS committee, we have developed an enhanced version of the RVQP. Our ED QI coordinator (research coordinator who also assists in the QI committee activities) sends out cases of return visits resulting in admission (automatically flagged by our electronic medical record), both to primary reviewers to analyze their own cases as well as to secondary reviewers (uninvolved physicians) with QI interest (whether formally trained or not). Unscheduled return visits are then analyzed with a root-cause analysis framework (patient/ provider/ system factors), in addition to other relevant elements, including ED overcrowding and social determinants of health. Reviewers are encouraged to think about QIPS interventions, which are then reviewed by the ED QI leads and QIPS committee for action (see appendix 1 for our return visit template).

3. Culture of safety encourages a constructive approach toward return visit audits.

The qualitative analysis of the Ontario RVQP revealed variations in local safety culture, which impacted the methods of analysis and the resulting attitude of frontline providers [6]. A centralized approach, where the ED medical director or manager completed the audits on behalf of the physician group, led to apprehension from frontline providers regarding what was perceived as performance reviews, due to historic medico-legal or punitive approaches to chart audits. Conversely, we and other centers use a distributed approach where every physician audits their own charts, with emphasis from ED leadership on identifying QI opportunities for the department as a whole. As a result, while our audits found that only 21% of return visits experienced an adverse event, with only 12% were attributed to cognitive lapses, 67% of audits were rated as useful by those completing them.

## Bottom-up

If only the left side of the model diagram were true, then ED return visit audits would be constrained by pre-existing QIPS culture and infrastructure. Fortunately, the engagement of frontline providers with return visit audits also feeds back into the development of QIPS capacity and a culture of safety, which is particularly relevant for centers without pre-existing QIPS infrastructure. EDs can use a variety of tools to build QIPS infrastructure and a culture of safety, but return visits are a shared experience for every provider in every ED, so audits are an important tool for EDs of any size.

4. Return visit audits provide experience that builds QI team and generates project ideas.

Return visit audits not only uncover safety issues and other QI learnings, but they can be part of building the QIPS infrastructure to address them [3, 7]: enlisting frontline providers to identify local priorities, highlighting patient stories to create a sense of urgency, and adapting interventions to the local context. A number of our QI projects have been motivated by return visit audits and helped build QIPS infrastructure, including: a protocol for repeating vital signs on discharge, a physician handover tool to improve transition of information and accountability, evidence-based order sets in response to return visits for alcohol withdrawal or undertreated sickle cell crises, and rapid follow-up clinics for addiction medicine or COVID. Anyone in our department is welcome to lead QI projects of their choosing, with the support of our QI committee (see our blueprint [3] for more project examples). A resident-driven model for return visit audits could also build up future QIPS infrastructure by training the next generation in QIPS methodology [8].

5. QI projects and academic dissemination develop and sustain a culture of safety.

QI projects can promote a culture of safety through their outcomes, but it is also important to consider their process. For example, a comparative study found that a smaller county hospital experienced greater improvement in safety culture than a larger university hospital, because the former had initiatives driven from the “bottom-up” by frontline physicians whereas the latter was “top-down” with little engagement31. In other words, larger academic centers need to be mindful they are not taking the initiative from frontline providers, and smaller community centers do not need large committees to improve patient safety [9].

This is where return visit audits play a crucial role—enlisting frontline staff in both identifying quality issues and designing solutions, both of which are important for improving safety culture. Many of our QIPS interventions have been shared through multiple publications [3] contributing to the science of QIPS and sustaining a culture of safety through academic dissemination, and a website to share articles and projects about Health informatics, Quality improvement and Patient safety (HiQuiPs; www.hiquips.com).

6. Sharing return visit audits breaks down silos and builds a broader culture of safety.

Return visit audits can promote a local safety culture, but these lessons are often not shared outside the department—a remnant of a medico-legal approach to safety issues that keeps EDs operating in their own silos. This can reinforce a divide between larger academic hospitals with QI infrastructure and smaller community hospitals without them.

We have developed and collaborated in novel strategies to break down these barriers. At the city-wide level, we collaborated with the Hospital for Sick Children on the development of routine audits [10], and with QIPS physician leads across the region (in both academic and community hospitals) to launch an Emergency Medicine Quality Improvement Digest [11]. At the provincial level, we have contributed to provincial webinars organized by Health Quality Ontario to share lessons to EDs across the province on how they can use their own return visits to build a culture of safety. At the national level, we have launched a new podcast on return visits and QIPS on the website Emergency Medicine Cases (emergencymedicinecases.com) targeted to all ED providers, irrespective of formal QIPS training.

## A practical assessment tool

Drawing from the above theoretical model, we have developed a practical assessment tool for any ED to assess their local strengths, limitations, and opportunities for QIPS growth, through the catalyst of return visit audits. Below are six questions progressing sequentially through the “top-down” and “bottom-up” sides of the model.

1. What is the current safety culture, and how can it be leveraged to expand QI infrastructure? For example, the ED Medical Director could support development of local QIPS infrastructure, including a QIPS Committee [3].
2. What is the current QI infrastructure and how can it support return visit audits, both quantitatively and qualitatively? For example, the QIPS committee could provide a return visit template and share skills with frontline providers to support return visit audits [5].
3. How is safety culture influencing return visits, and can this be improved? For example, using distributive methods and emphasizing system-wide quality improvement instead of individual performance [6].
4. How can return visit audits be used to train frontline providers in QI methodology, develop their skills, and build local QIPS infrastructure? For example, establish a formal QI team, train secondary reviewers, and formalize change ideas into specific QI projects [3, 8].
5. How can QI projects be used to develop and sustain a culture of safety? For example, use QI projects in conjunction with formal departmental support to increase visibility and uptake of local QIPS projects, and to share more broadly though academic dissemination [3].
6. How can sharing return visit audits break down silos and build a broader culture of safety? For example, collaborate with other EDs or hospital departments to break down silos and normalize the discussion of return visits [10].

## Supplementary Information

Below is the link to the electronic supplementary material.

Supplementary file1 (DOCX 15 KB)
