# Supplementary material for: Return visit audits, quality improvement infrastructure, and a culture of safety: a theoretical model and practical assessment tool
Source: CJEM. 2023 Jun 15;25(8):649–52. doi: 10.1007/s43678-023-00539-6 (PMC10425292; doi:10.1007/s43678-023-00539-6)
Supplement: Supplementary file 1 — Supplementary file1 (DOCX 15 KB) [file 43678_2023_539_MOESM1_ESM.docx]

**Return visit audit template**

**Providers**

1. Were you involved in this patient’s care: Yes / Second-level reviewer
2. Was a resident/trainee involved: Yes / No

**Case summary**

1. Was this a sentinel event: myocardial infarction / subarachnoid hemorrhage / pediatric sepsis / no
2. Summary of first visit:
3. Summary of return visit:
4. Type of adverse event/quality issues:

diagnostic issue / management issue / medication adverse event /

unsafe disposition decision / procedural complication / suboptimal follow up

1. Impact: none / mild / moderate / severe / death

**Analysis**

1. Were any adverse events/quality issues identified: Yes / No
2. What was the most appropriate theme applicable to this case:

Patient risk profile / Elder care / Documentation / Physician cognitive lapses

Left against medical advice, Left without being seen / Vital signs

High risk medications or interactions / Handover/communication

Radiology / Imaging testing availability /

Discharge planning/community follow up / Other

1. Was overcrowding an important factor that led to this specific case’s outcome: Yes / No
2. Were social determinants of health (SDOH)—including but not limited to societal racism, precarious housing or poverty—were important factors that led to this specific case’s outcome: Yes / no
3. If you answered yet to overcrowding or SDOH, or there are additional themes, please expand on your answer here:

**Quality improvement**

1. Were there any educational pearls, system-wide operational or quality improvement areas identified in this case: Yes / No
2. Potential actions for quality improvement:
3. Intervention type:

forcing function / automation, computerization / reminder, checklist

rules, policies / education, training

**Awareness**

1. Do you want to receive the second level reviewer’s analysis: Yes / No
2. Should our ED nursing leadership be made aware of this case: Yes / No
3. Should the consulting service leadership be made aware of this case: Yes / No
